# Evaluating the Efficacy of Time for Living and Caring: An Online Intervention to Support Dementia Caregivers’ Use of Respite

**DOI:** 10.1093/geroni/igae043

**Published:** 2024-04-26

**Authors:** Eli Iacob, Michael Caserta, Gary Donaldson, Catharine Sparks, Alexandra Terrill, Amber Thompson, Bob Wong, Rebecca L Utz

**Affiliations:** College of Nursing, University of Utah, Salt Lake City, Utah, USA; College of Nursing, University of Utah, Salt Lake City, Utah, USA; College of Nursing, University of Utah, Salt Lake City, Utah, USA; College of Health, University of Utah, Salt Lake City, Utah, USA; College of Health, University of Utah, Salt Lake City, Utah, USA; College of Social and Behavioral Sciences, University of Utah, Salt Lake City, Utah, USA; College of Nursing, University of Utah, Salt Lake City, Utah, USA; College of Social and Behavioral Sciences, University of Utah, Salt Lake City, Utah, USA

**Keywords:** ADRD/dementia, Caregiver, Clinical trial, Digital health or M-health interventions, Time-use

## Abstract

**Background and Objectives:**

Respite, defined as time away from caregiving, is the most requested type of caregiver support. Time for Living and Caring (TLC) is a virtual coaching “app” that helps caregivers schedule and plan their respite time-use. The objectives of this analysis are: (1) to assess the efficacy of the TLC intervention on respite time-use and on caregiver well-being and (2) to identify the key features of the intervention that serve as the likely mechanism of action.

**Research Design and Methods:**

A sample of dementia caregivers (*n* = 163, 79% female, 84% White, 6% Hispanic, average age 62) were randomized into one of two intervention delivery methods. Intervention efficacy was evaluated using pre/post-comparisons of respite time-use and an additive “dosing” model that estimated unique parameters associated with the exposure to each specific intervention component.

**Results:**

Both immediate and delayed-attention groups reported increased respite time. They also improved in their ability to plan and perceive benefit from their respite time-use over the 16-week intervention period. At 8 weeks, the immediate group did not change in anxiety, whereas the delayed group worsened (*p* < .001). At 16 weeks, the groups were similar in their anxiety levels. By the 20-week follow-up period, when neither group had access to TLC, both experienced an increase in anxiety.

**Discussion and Implications:**

TLC is a promising intervention that may support caregivers’ well-being, by helping them schedule and plan their respite to maximize its benefit. The provision of weekly coaching seems to be the intervention component (mechanism) associated with caregiver outcomes.

**Clinical Trial Registration:**

NCT03689179

**Translational Significance:** Caregivers report feeling guilty, dissatisfied, or disappointed with respite because they did not know what to do or felt they wasted their time off. Time for Living and Caring (TLC) is a self-administered, app-delivered intervention to coach caregivers on how to plan and maximize respite time-use. Use of TLC is associated with increased respite time and greater satisfaction with respite time-use. The goal-setting and goal-review features appear to be the mechanism of action, associated with a maintenance (no increase) of anxiety over time. The TLC intervention may provide a cost-efficient and scalable solution to maximize benefits of respite, the most needed and desired caregiver support.

Currently, 6.7 million Americans are living with Alzheimer’s disease and related dementia—expected to grow to 88 million by 2050. More than 11 million Americans provide care for a family member or friend with dementia, at an estimated value of $340 billion annually (Alzheimer’s Association, 2023). These caregivers perform instrumental and technical care, often without adequate support or training and usually without any compensation (AARP & NAC, 2020). Caregivers often experience physical and mental health declines, report financial hardships, and make notable personal sacrifices as a result of the caregiver role (Adelman et al., 2014; Schulz et al., 2020). Dementia caregivers report exceptionally high levels of daily stress (Kasper et al., 2015), given the challenging and extended nature of the disease (Scheltens et al., 2016). Furthermore, due to demographic shifts associated with reduced fertility, increasing divorce rates, and greater numbers of women in the workforce (Carr & Utz, 2020), the number of available family caregivers is not keeping pace with the numbers of older adults needing care. Likewise, shortages in the direct-care workforce also increase our reliance on family caregivers. Thus, establishing ways to support existing family caregivers, especially dementia caregivers, who may be at greatest risk for burnout and stress, is critical for our nation’s public health and economic well-being.

A number of caregiver interventions have been developed over recent decades (Czaja et al., 2006; Meyer et al., 2018; Schulz et al., 2003), addressing self-care, safety, social support, emotional well-being, management of behavioral problems, skills training, telephone-based support, behavior modification, family therapy, computerized telephone communication, coping methods, and support groups (Badr et al., 2015; Bourgeois & Burgio, 1996; Chi et al., 2015; Cooke et al., 2001). Most caregiver support programs have high participant satisfaction but only produce modest benefits for caregiver outcomes (Schulz et al., 2002; Whitlatch & von Eye, 1991). Surprisingly, very few of the existing interventions focus on respite (Gaugler, Jarrott, et al., 2003; Gaugler, Zarit, et al. 2003; Gaugler et al., 2016).

*Respite* is defined as time away from caregiving (Zarit et al., 2017). Respite is provided by adult day programs, in-home respite providers, institutional settings, or through the tag-teaming or shared arrangements that families, friends, and neighbors set-up to ensure that the care-recipient’s needs are fully taken care of and that the primary caregiver gets an occasional break. Respite is commonly identified as the most needed and desired type of services for caregivers (Lund et al., 2009; Utz et al., 2012) and has been said to be the most promising ways to maintain and enhance caregiver well-being (ARCH, 2015). When scheduled regularly and in sufficient doses (Zarit & Greene, 1998; Zarit et al., 2014), respite gives caregivers time to address their own health, maintain social and family relationships, and pursue other aspects of their daily lives that they may have neglected since becoming a caregiver (Evans, 2013).

Past research on the effectiveness of respite finds only moderately positive, and often inconsistent or mixed results, on its overall benefit to caregivers (Kosloski & Montgomery, 1993; Sorensen et al., 2002; Zarit & Greene, 1998; Zarit et al., 2017). From an intervention standpoint, there exists a need to understand how to make respite more effective. Furthermore, with national and state funding levels for respite programs being limited and/or relatively flat (Rose et al., 2015), there is a need to enhance the value that caregivers get from respite because the amount of respite available to a caregiver is often quite limited.

Prior research found that almost half of caregivers said they were dissatisfied with their respite (Lund et al., 2009). Many reported “wasting” time instead of using their respite as a reprieve from their role as vigilant caregiver or to pursue activities that are personally meaningful or rewarding. Time-use research has long confirmed that congruence between desired and actual time-use is a predictor of overall life satisfaction and well-being (Seleen, 1982). An expert panel assembled by ARCH (a federally funded national respite network and resource center) summarized the state of research on respite and provided guidelines for future research; their recommendations included finding ways to make respite more effective, including a greater focus on the caregivers’ time-use during respite (ARCH, 2015).

## TLC Intervention

**Figure 1.**
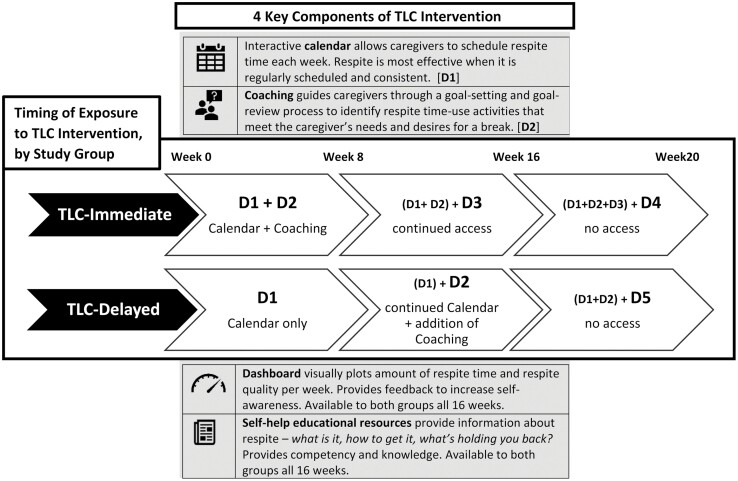
Description of four key components of the Time for Living and Caring (TLC) Intervention and timing of exposure by study group. The TLC-Immediate Group received access to both the calendar (D1) and coaching (D2) for the first 8 weeks, followed by continued access to these resources (D3) for the second 8 weeks. The TLC-Delayed group received access to the calendar (D1) for first 8 weeks, followed by the addition of the coaching (D2) for the second 8 weeks. Both groups had access to the dashboard and educational resources throughout the 16-week intervention period. Both groups lost access to the TLC intervention after week 16 and were followed up for research purposes until week 20 (D4, D5).

The Time for Living and Caring (TLC) intervention is a 16-week online, self-administered intervention designed to help caregivers schedule, plan, and maximize the benefit of their respite time-use. As shown in Figure 1, TLC has four key components: (1) a *calendar* to schedule respite, (2) *virtual coaching* to help caregivers plan their respite time-use, (3) a *dashboard* to visually track respite quantity and quality, and (4) *educational and resource pages* related to respite and caregiver well-being broadly.

The TLC intervention is premised on the belief that caregivers would receive greater benefit from respite if (1) they regularly scheduled respite (Zarit & Greene, 1998; Zarit et al., 2014) and (2) their time-use represented activities that were personally meaningful and desired (Lund et al., 2009; Utz et al., 2012). The TLC calendar was designed to assist with the former (i.e., scheduling respite), whereas the TLC coaching feature was designed to achieve the latter (i.e., planning respite time-use). The coaching module worked in tandem with the digital calendar. Coaching consisted of weekly automated prompts based on the principles of the “selective optimization with compensation” (SOC) theory of aging (Baltes et al., 1996; Freund & Baltes, 2002). Prompts guided caregivers to develop individual time-use goals that were SMART (specific, measurable, achievable, relevant, and time-based; Bovend’Eerdt et al., 2009), by first having them *select* a desired set of goals, and then asking whether they needed to *optimize* or refine those goals based on personal constraints or opportunities (e.g., not enough time or too costly, daughter in town and able to provide extended break). By going through this process weekly, participants practiced *compensation*, when they were prompted to revise the goal if they were not able to accomplish it during the respite time available. The dashboard visually displayed their progress and successes in scheduling and planning respite time-use, whereas the education and information pages provided self-help support resources including links to find local respite providers, fillable worksheets that caregivers could leave with a respite provider coming into the house, instructions on how to have a family meeting to ask for assistance from others, and a short recorded meditation script that helped caregivers let-go of caregiving stresses prior to scheduled respite. Together, all four components of the intervention provided support with the goal of improving their respite time-use, but the calendar and coaching features, which are delivered similarly every week and call for individual interaction via the app, represent the key intervention components that are hypothesized to drive behavior change and produce meaningful effects on caregiver well-being.

Participants were randomized into one of two delivery groups: *TLC-Immediate* received access to the calendar and coaching for 16 weeks. *TLC-Delayed* received a staggered introduction to the intervention, receiving access to the calendar for 8 weeks, followed by the addition of weekly coaching for the second 8 weeks. Figure 1 uses the letter “D” to describe the different timing and length of exposure to these key intervention features. TLC-Delayed is reminiscent of the “waitlist” condition in a traditional waitlist-control study design a common methodology used in clinical trials of behavioral interventions (Terrill et al., 2018). TLC adopted a modified waitlist to provide some attention during the initial “waitlist” period (first 8 weeks). This decision was based on an assumption that caregivers should first establish a regular respite schedule before they can successfully engage in time-use planning about what specific activities they want to do during their scheduled respite (Lund et al., 2009). Those assigned to TLC-Immediate could simultaneously and iteratively practice scheduling (calendar) and planning their respite time-use goals (coaching) for the entirety of the intervention. Behavioral interventions typically recommend a minimum of 6–8 weeks to achieve lasting behavior changes (Gitlin & Czaja, 2015). Both TLC-Immediate and TLC-Delayed had access to the key intervention features for a minimum of 8 weeks and up to 16 weeks, likely providing sufficient exposure to initiate behavior change and produce measurable effects on caregiver outcomes. The TLC intervention and study design were cocreated through a collaborative, community-engaged process, whereby the research team, technology developers, and media producers worked closely with a community advisory board (composed of current and former caregivers, respite providers, and cultural leaders) to ensure that the intervention and research study were culturally responsive, relevant, and reflective of the needs, wishes, and identities of diverse caregivers (Utz et al., 2023).

## Current Study and Research Questions

The purpose of this pilot study is to evaluate the initial efficacy of the TLC intervention. We define efficacy in terms of caregivers’ respite time-use (i.e., number of hours, satisfaction with time-use), as well as their anxiety. We selected anxiety as our chosen outcome, as this is a measure of well-being associated with the daily life stresses of caregiving (del-Pino-Casado et al., 2021) and that has been responsive to time-use interventions in other contexts (Ping & Xiaochun, 2018).

Currently, TLC is in Stage 1B (pilot-testing), according to the NIA Stage Model of Behavioral Intervention Development (Onken et al., 2014). A critical focus of pilot-testing is to assess feasibility and usability of the intervention and research procedures (to be reported elsewhere), as well as to identify and evaluate the mechanism underlying an intervention’s effect (Gitlin & Czaja, 2015). The TLC research study used a fully powered pilot sample, a modified waitlist-control randomized study design, and an analytic technique where we estimated parameters for each specific intervention component (i.e., calendar and coaching feature) that may affect caregiver outcomes. Our approach is able to identify not just the outcomes indicating intervention efficacy but also how specific intervention features serve as the theoretical and empirical mechanism underlying intervention efficacy.

## Method

### Data and Study Design

Data come from the TLC study, a pilot-test of the TLC intervention. Data include self-reported survey-based questionnaires collected at baseline (pre-intervention), 8 weeks, 16 weeks (post-intervention), and 20 weeks (maintenance of effect, follow-up). All study procedures were preregistered in Clinical Trials (NCT03689179) and approved by the University of Utah Institutional Review Board (IRB_00120589).

### Sample

The TLC sample included those who (a) self-identified as primary caregiver to someone with Alzheimer’s disease or dementia, (b) lived in the same house as the person with dementia, (c) were English speaking (because the intervention was developed in English only), (d) were over the age of 18, and (e) had access to and/or interest in using respite at least once a week for a minimum of 4 hours. This final criterion was loosely applied during recruitment and screening because the study was done during the coronavirus disease 2019 (COVID-19) pandemic when many formal respite services were not consistently available; thus, in practice, this eligibility criterion emphasized caregivers’ interest in taking a break of any kind, rather than being a current user of formal respite services.

**Figure 2.**
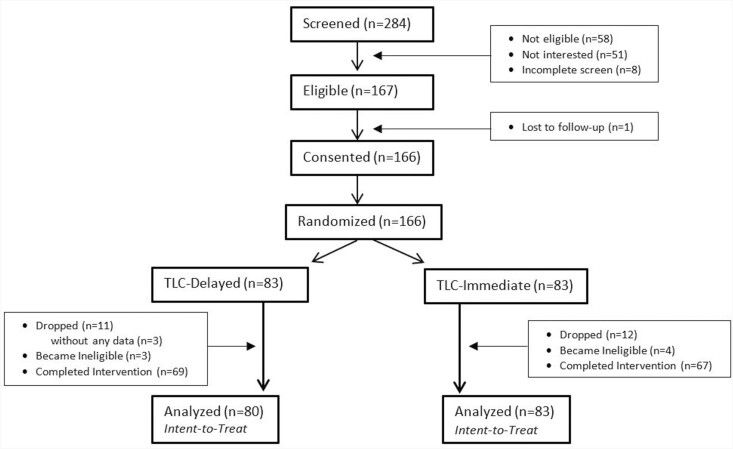
CONSORT diagram for the Time for Living and Caring (TLC) Study.

Potential participants were identified using community-engaged recruitment methodologies, including the use of community health workers and cultural leaders as recruiters, word-of-mouth referrals from multiple local and national community partners (e.g., Caregiver Support Program through AAAs, state chapters of Alzheimer’s Association and AARP, and respite providers as identified by ARCH National Respite Resource Network) and from a clinical database from a single cognitive disorders clinic in Utah that identified a care partner for each patient who was assessed for dementia. (For more detailed information about the recruitment strategies used in the TLC study, including the time and cost of the multipronged recruitment strategies, see Sparks et al., 2024.) Figure 2 provides the CONSORT flow diagram, describing the screening, enrollment, and participation numbers associated with the TLC sample. The 58 referrals deemed “ineligible” included those who were not caring for someone with dementia (*n* = 37), not considered a “primary” caregiver (*n* = 34) or not living with the person with dementia (*n* = 6); only 1 was not eligible because of not having access to any form of respite or informal break from caregiving activities (*n* = 1). A total of 163 dementia caregivers comprised the sample used for this analysis.

### Measures

#### Independent variable

The key independent variable identifies membership into one of the two intervention groups: TLC-Immediate (*n* = 83) versus TLC-Delayed (*n* = 80). Participants were randomized upon enrollment using a predetermined schema of permuted blocks (size 2, 4, or 6) to ensure that the researchers were blinded to treatment allocation and that each block maintained a 1:1 sample size across treatment groups. Group membership defines the timing and length of exposure to each intervention component (i.e., dose of intervention); refer to Figure 1.

#### Dependent variables

Several variables were used to measure intervention efficacy. Measures related to caregivers’ respite time-use were obtained from five self-report survey questions, collected pre- and post-intervention: (1) “How much respite do you typically get per week?” (in hours per week), (2) “How much respite time would you like to get?” (in hours per week), (3) “Is your respite time scheduled in advance?” (always, sometimes, rarely, never, 4) “I am getting enough respite time” (strongly agree, agree, neutral, disagree, strongly disagree), and (5) “I am happy with what I chose to do during my respite time?” (strongly agree, agree, neutral, disagree, strongly disagree). These measures were created specifically for the TLC study; they are intended to be analyzed separately.

The second variable used to measure intervention efficacy was anxiety, a measure of caregiver well-being that has been responsive to other types of interventions (Cooper et al., 2007). Anxiety was measured with the PROMIS Anxiety short-form questionnaire for adults (α = 0.943), a 7-item additive scale that standardizes the distribution of anxiety-related symptoms on a population-distribution with a mean of 50 and standard deviation of 10 (Pilkonis et al., 2011): “During the past 7 days, I felt nervous, anxious, fearful, uneasy, tense, worried, unable to focus on anything other than my anxiety,” each assessed with a five-category response (never, rarely, sometimes, often, always). If a participant was missing more than 30% of the items (i.e., 2 or more items on the anxiety questionnaire), the scale score was coded as missing. Under these criteria, the full TLC sample has a calculated score for anxiety.

#### Demographic covariates

Multivariable analyses controlled for *caregiver relationship* (spouse vs nonspouse), *sex* (female vs male), *race* (White vs non-White), and *education* (completed college or more vs less than college). These four dichotomous variables represent the most parsimonious way to capture the sociodemographic characteristics associated with social disparities in health, normative caregiving roles and expectations, and mental health experiences more specifically.

### Analysis

All questionnaire and study tracking data were stored in Research Electronic Data Capture (REDCap) (UL1TR002538 NCATS/NIH). All data were cleaned and analyzed using SPSS, version 28.0 (IBM Corp.). Missing Values Analysis was conducted to explore if there were systematic patterns of missing data in the study overall. Out of the 163 participants, 151 (92.6%) had monotonic missingness of timepoints, and 12 (7.4%) had nonmonotonic missingness. Based on the low number of nonmonotone patterns, we did not employ pattern mixture models or multiple imputations to the TLC study/sample overall.

Descriptive statistics—including frequency counts, percent distribution, range, mean, median, and standard deviation—were calculated for all variables. Descriptive statistics are reported for the full TLC sample (*n* = 163), and then for the TLC-Immediate or TLC-Delayed subgroups (*n* = 83, *n* = 80, respectively). We conducted independent samples statistics (chi-square, Fisher’s exact test, Student’s *t* test, and Mann–Whitney tests), as appropriate, to statistically compare TLC-Immediate versus TLC-Delayed for baseline equivalency. We also used chi-square, paired-samples Student’s *t* tests, Wilcoxon Sign rank tests, and ANCOVA models to statistically evaluate individual-level change between pre- and post-intervention assessments. All alphas were set at 0.05.

We used generalized linear categorical dose-response models where the effect of each intervention component, duration of exposure, and maintenance over time on caregiver anxiety were estimated. The regression defined and estimated five separate parameters: D1, access to calendar; D2, access to weekly coaching; D3, continued exposure to the full intervention (for TLC-Immediate group only); and two separate components (D4, D5) to capture potential maintenance of intervention effects once the intervention is “turned off” (weeks 17–20). D4 reflects the experience of TLC-Immediate group and D5 is associated with the TLC-Delayed group, estimated separately assuming that the maintenance effect may be different since intervention exposure and timing during weeks 1–16 were different across groups. As shown in Figure 1, each group and each timepoint has a linear combination of parameters that describe the effect of overall intervention exposure: the TLC-Immediate group is calculated by a linear combination of parameters D1, D2, D3, and D4, whereas the overall effect of TLC-Delayed would be calculated by a linear combination of D1, D2, and D5 parameters.

Using this model, parameter estimates can be positive or negative. The approach treats time categorically, thereby not imposing any specific linear pattern to change over time. The null hypothesis of no benefit of having access to the calendar for 8 weeks (D1) is defined as D1 = 0. The null hypothesis for no added benefit of the coaching component (D2) is D2 = 0. The null hypothesis of receiving no benefit of having an additional 8 weeks of access of calendar and coaching for the TLC-Immediate group is D3 = 0. Finally, the null hypotheses that there is no further benefit between weeks 17–20 when the TLC intervention was no longer available is D4 = 0 (for TLC-Immediate) and D5 = 0 (for TLC-Delayed). Statistical significance was set to alpha < 0.05.

A second model assessed the impact of covariates, including relation to patient (spouse or nonspouse), gender (female vs male reference group), race (non-White vs White as reference group), and education (college and above vs noncollege as reference group). Log likelihood with Chi-square difference compared unadjusted and adjusted models.

#### Power analysis

We assumed clinically relevant small to medium effect sizes, corresponding to a 1.0–1.4 mean change in PROMIS anxiety scores. Power and sensitivity analyses were computed with GPower 3.1 (Faul et al., 2009), finding that a total sample of 134 would be sufficient to achieve power of greater than 90% to detect effect sizes for three total timepoints and 0.3 correlation between measures. The current over-recruitment fully powered sample of 163 accounts for attrition (~15% of baseline TLC sample). All multivariable analyses utilized multilevel mixed-effects regression modeling under an intent-to-treat assumption and utilizing restricted maximum likelihood, such that all available data were utilized.

## Results

### Sample Demographics

The TLC sample included dementia caregivers who were, on average, 61.7 years of age (standard deviation 13.0, min 20, max 92). They were primarily spouse or partner (68.1%) or adult child to care-recipient (24.1%). Most were female (78.9%), White (82.5%), non-Hispanic (90.4%), married or living with partner (83.7%), had a college education (89.7%), and incomes greater than $50,000 annually (73.6%). There were no significant differences, suggesting baseline equivalency across the two groups. Refer to Supplementary Table 1 for complete demographics of TLC sample and study-group subsamples.

### Respite Time-Use

As shown in Table 1, participants in both TLC-Immediate and TLC-Delayed reported increased respite time and increased respite satisfaction following exposure to the TLC intervention. As there were no significant baseline differences, results for full TLC sample are reported here. Caregivers increased respite hours per week: baseline = 8.58 (*SD* 11.59); post-intervention = 13.28 (11.74). An ANCOVA model estimated the number of post-intervention respite hours controlling for baseline respite use, showing a post-intervention average of 9.53 hours [6.15, 12.90] with an increase of 0.46 hours [0.25, 0.66] for each hour of respite that a caregiver had at baseline (*p < *.001). Thus, a caregiver with 10 hours of respite at baseline is predicted to have 9.53 + 10 × 0.46 = 14.09 hours of respite at the end of the intervention.

**Table 1.** Self-Reported Respite Time-Use and Anxiety at Pre-Intervention (Baseline) and Post-Intervention for the TLC Sample, With Comparison of the TLC-Immediate and TLC-Delayed Subsamples

Outcomes	Full TLC Sample	TLC-Immediate	TLC-Delayed	*p* Value^a^
*Typical number of respite hours per week,* Mean (*SD*)				.54
Pre	8.33 (9.95)	8.77 (8.89)	7.81 (11.01)	
Post^b^	13.28 (11.74)^**^	13.44 (12.64)^*^	13.11 (10.82)^**^	
*Ideal number of respite hours per week,* Mean (*SD*)				.58
Pre	18.44 (19.33)	20.14 (12.99)	16.67 (24.23)	
Post^b^	22.00 (13.64)^*^	22.19 (15.15)	21.73 (11.92)	
*Respite time is scheduled in advance*, % Always/Sometimes				.09
Pre	57.8%	62.7%	52.9%	
Post^b^	89.6%^**^	89.5%^**^	89.7%^**^	
*Getting enough respite*, % Strongly Agree/Agree				.09
Pre	9.6%	10.5%	8.8%	
Post^b^	41.7%^**^	31.4%^**^	51.5%^**^	
*Happy with what I choose to do during respite*, % Strongly Agree/Agree				.77
Pre	44.4%	46.3%	42.7%	
Post^b^	73.4%^**^	73.1%^**^	73.5%^**^	
*Anxiety score*, Mean = 50 (*SD* = 10)				.33
Pre	54.38 (9.54)	55.52 (10.03)	53.57 (9.02)	
Post^b^	54.65 (9.10)	56.54 (9.54)	52.75 (8.28)	

*Notes*: TLC = Time for Living and Caring. The sample used to calculate descriptive statistics for this table was restricted to those who had data at both pre- and postassessment.

^a^Baseline mean differences were assessed with independent samples *t* test and Mann–Whitney *U* tests, as appropriate. There are no statistically significant differences, *p* > .05 for all variables, suggesting baseline equivalency of the TLC-Immediate and TLC-Delayed subgroups.

^b^Change over time was assessed with ANCOVA (postvalue conditioned on baseline value)—see text—and Wilcoxon sign tests, as appropriate. For categorical variables, the entire 5-point Likert scale was used. However, we report here Yes/Sometimes and Strongly Agree/Agree descriptive statistics for ease of interpretation. Post-intervention was measured at 16 weeks, with exception of desired/ideal hours, which were measured at 20 weeks.

^*^ *p < *.05.

^**^ *p* < .01.

Caregivers also reported modest increases in the amount of respite they would like to have in a typical week, from an average of 19.87 hours (*SD* = 22.59) at baseline to 21.96 hours (*SD* = 13.59) post-intervention. ANCOVA models controlling for baseline levels of desired respite estimated post-intervention desired hours to be 17.90 [13.70, 21.89] plus an increase of 0.24 hours [0.11, 0.37] for each desired hour at baseline (*p < *.001). Therefore, a caregiver desiring 20 hours of respite at baseline is predicted to have 17.90 + 20 × 0.24 = 22.70 hours of desired respite at the end of the intervention.

Caregivers reported a substantial increase in scheduling respite, with 27.6% stating they regularly scheduled, 29.4% sometimes scheduled, and 40.5% rarely scheduled their respite at baseline, compared to 42.2% regularly scheduling, 47.4% sometime scheduling, and 6.7% rarely scheduling at post-intervention (*Z* = 5.56, *p < *.001). Caregivers also reported increased respite satisfaction: only 10.5% agreed or strongly agreed that they got enough respite at baseline, compared to 41.5% at the post-intervention period (Wilcoxon sign test on full Likert scale, *Z* = 7.709, *p < *.001). Similarly, a significantly greater number of caregivers were happy with what they chose to do during respite at the end of the intervention compared to before (73.4% post-intervention compared to 43.0% at baseline; *Z* = 5.965, *p < *.001).

### Caregiver Anxiety

Table 2 displays the mixed-effects regression results used to estimate the dose-response effects associated with the timing and exposure of the key TLC intervention components on caregiver anxiety. Because the covariate-adjusted Model 2 was superior to the unadjusted Model 1 (χ^2^ (4) = 151.68, *p < *.001), results from Model 2 are reported below. In terms of covariates, females reported significantly higher anxiety than males (3.76 [0.71, 6.81]), but there were no significant differences in anxiety by relationship type (*p = *.90), education (*p = *.07), or race (*p *= .50). Sensitivity models that included sex × parameter interaction terms were insignificant and therefore we retained and reported the more parsimonious sex-adjusted models.

**Table 2.** Categorical Dose-Response Models Predicting Changes in Anxiety Associated With Each Component of the TLC Intervention

Predictor variables	Model 1				Model 2			
	Estimate	*SE*	*p* Value	[95% CI]	Estimate	*SE*	*p* Value	[95% CI]
*Fixed effects*								
Intercept	54.73	0.69	<.001	[53.38, 56.08]	52.12	2.30	<.001	[47.56, 56.67]
Intervention component^a^								
D1 (calendar)	3.31	0.76	<.001	[1.82, 4.80]	3.27	0.77	<.001	[1.75, 4.79]
D2 (coaching)	−3.17	0.77	<.001	[−4.69, −1.65]	−3.22	0.79	<.001	[−4.76, −1.67]
D3 (continued access)	0.78	0.77	0.314	[−0.74, 2.30]	0.82	0.77	0.286	[−0.69, 2.33]
D4 (no access)	1.66	0.85	0.051	[−0.01, 3.33]	1.67	0.84	0.048	[0.02, 3.31]
D5 (no access)	2.26	0.79	0.005	[0.70, 3.81]	2.16	0.81	0.008	[0.57, 3.75]
Demographic covariates								
Female					3.76	1.54	0.016	[0.71, 6.81]
Spouse					−0.18	1.41	0.901	[−2.96, 2.61]
College					−2.32	1.25	0.066	[−4.80, 0.15]
White					1.28	1.89	0.500	[−2.46,5.03]
*Random effects*								
Residual variance	23.16	1.65	<.001	[20.13, 26.64]	22.62	1.64	<.001	[19.62, 26.08]
Intercept variance	53.50	6.82	<.001	[41.67, 68.68]	52.81	6.93	<.001	[40.84, 68.29]
Log likelihood	3,671.47				3,519.79			
Df	8				12			

*Notes*: CI = confidence interval; D1 = access to calendar. D2 = access to coaching (initial assessment, goal-setting, goal review); D3 = continued access to TLC intervention (2nd 8 weeks, TLC-Immediate only); D4 = no access to TLC intervention; maintenance of effect between weeks 17–20 (TLC-Immediate only); D5 = no access to TLC intervention; maintenance of effect between weeks 17–20 (TLC-Delayed only); *SE* = standard error; TLC = Time for Living and Caring; χ^2^ difference between models: 151.66, *p* < .001.

^a^Exposure to the TLC intervention is modeled in terms of its major components, with each component representing a “dose” of the intervention; refer back to Figure 1.

Having access to the TLC calendar (D1) significantly increased anxiety by 3.23 [1.75, 4.79], while having access to the coaching feature of the TLC intervention (D2) significantly decreased anxiety by −3.22 [−4.76, −1.67]. Based on a population standard deviation of 10, as is common for all PROMIS measures (Pilkonis et al., 2011), these two parameter estimates are equivalent to an effect size of about 0.32, a moderate effect size. D1 is a negative effect associated with an increase in anxiety, whereas D2 is a positive effect associated with a reduction in anxiety. Drawing on the additive assumption of the modeling approach, having access to D1 + D2 simultaneously would, therefore, mitigate the effects of each component alone, as determined by custom contrast calculated by the addition of the two estimated parameters for D1 and D2 0.051 [−1.086, 1.188]; this is not statistically different than 0 and can be interpreted as a maintenance or “no change” in anxiety in the TLC-Immediate group at 8 weeks. Continued access to TLC (D3, only available to TLC-Immediate) was associated with no statistically significant change in anxiety over time (D3 = 0.82 [−0.59, 2.33]). For both groups, anxiety significantly increased during the time when intervention access was removed, measured at 20 weeks. In TLC-Immediate, D4 = 1.56 [0.02, 3.31], with estimated end-of-study anxiety defined by D1 + D2 + D3 + D4 = 2.536 [1.027, 4.046]. In TLC-Delayed, D5 = 2.16 [0.57, 3.75], with estimated end of study anxiety by D1 + D2 + D5 = 3.032 [0.981, 5.082].

**Figure 3.**
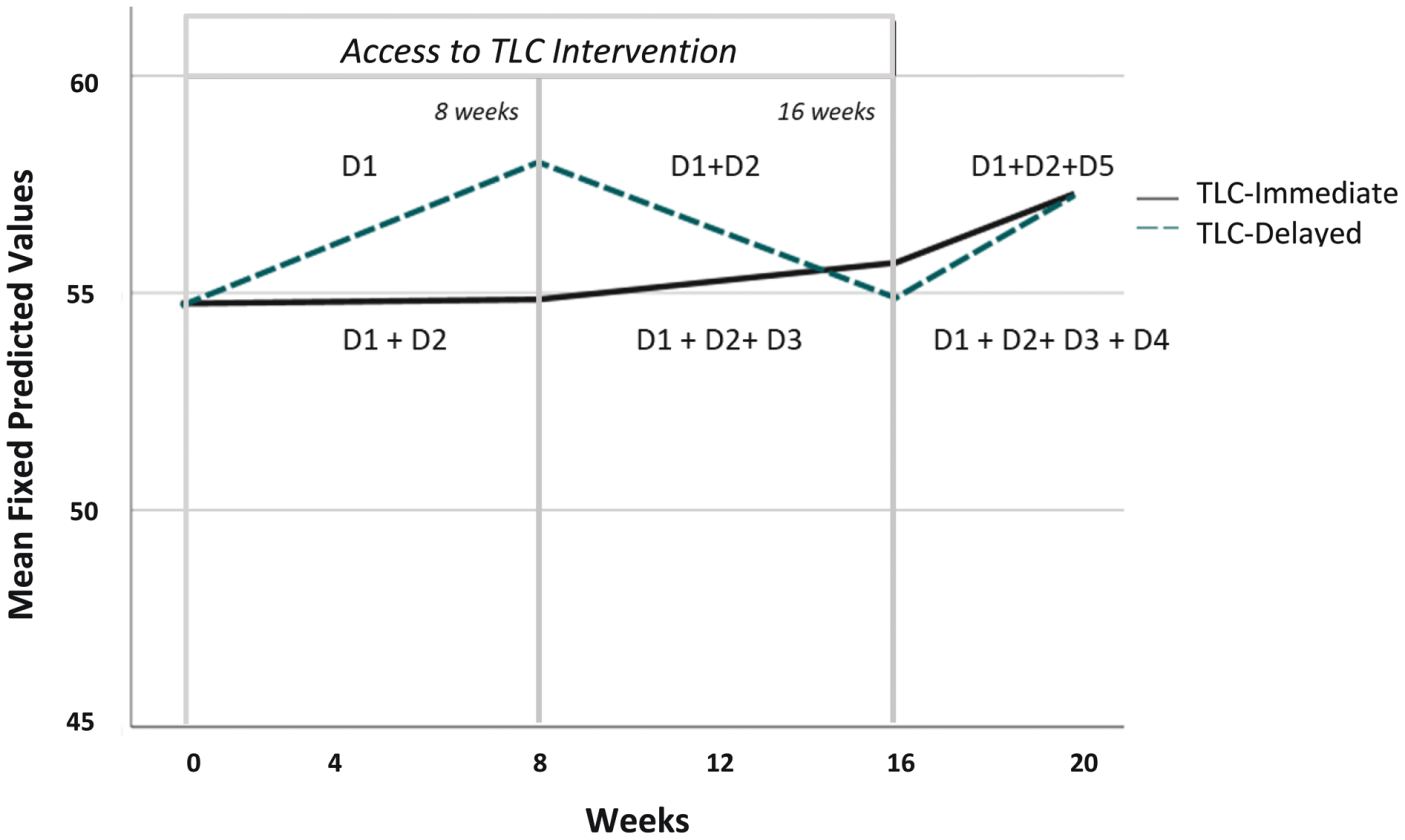
Mean Predicted Anxiety Scores, plotted by time (in weeks) for TLC-Immediate and TLC-Delayed groups. TLC = Time for Living and Caring. Each time point represents exposure to different components (D = doses) of the TLC intervention, as noted below and as described in Figure 1.

Figure 3 plots predicted means for anxiety (as calculated from Model 2 of Table 2) at baseline, 8, 16, and 20 weeks, along with the associated description of which intervention components (dosing) each study group was exposed to during the 16-week intervention period and the 20-week study. Figure 3 substantiates the findings from Table 2, providing further evidence that the weekly coaching component of TLC (D2) is the “dosing” or intervention feature most associated with improvement and/or maintenance of well-being (anxiety) over time.

## Discussion

This manuscript describes the TLC intervention and the research design used to evaluate its efficacy. TLC was designed to help dementia caregivers schedule and plan respite time-use in response to previous research showing that a majority of caregivers reported feeling guilty, dissatisfied, or disappointed with respite because they did not know what to do or felt they wasted their time off (Lund et al., 2009). TLC’s interactive calendar and automated coaching guide caregivers through a weekly goal-setting and goal-review process (Bailey, 2019) that encourages them to set SMART time-use goals (Bovend’Eerdt et al., 2009) that maximize the benefits of respite, whether they have limited or ample respite hours available.

There is substantial evidence for the efficacy of the TLC intervention, especially in terms of creating and sustaining behavior change related to respite time-use. Pre–post comparisons revealed that caregivers increased their amount of respite hours over the course of the 16-week intervention period. Caregivers also reported much greater likelihood of scheduling their respite and being satisfied with what they chose to do during respite when comparing pre–post responses. These are important outcomes in their own right, especially because previous research states that respite needs to be scheduled in advance and provided in sufficient doses in order to be effective (Zarit et al., 2017). In addition, time-use research has consistently found an empirical association between well-being and time-use (National Research Council, 2013), especially when people are able to do the types of activities they choose or desire to do (Utz et al., 2012).

To assess intervention efficacy, we also modeled TLC’s effect on caregiver anxiety. On average, and across two different exposure groups, caregivers generally maintained their baseline anxiety levels at the end of the intervention period (week 16), and experienced a significant increase when they no longer had access to the intervention (weeks 17–20). More specific findings, afforded by our nontraditional analytic approach that estimated parameters associated with the unique timing and exposure to specific components of the intervention, revealed that “coaching” is likely the most important intervention feature. When caregivers were exposed to the coaching feature, anxiety levels either decreased (TLC-Delayed) or stayed the same (TLC-Immediate) over time. For TLC-Delayed, anxiety levels initially increased when they were exposed to the calendar alone, yet decreased when they had added exposure of the coaching feature. It is possible that having access to the calendar only (weeks 1–8 for TLC-Delayed) may have heightened awareness that they did not have enough respite time or that they lacked a consistent respite schedule from week to week. Likewise, not having any TLC support (during weeks 17–20 for both groups) was associated with marked increases in anxiety, showing that there is no maintenance of the effect on anxiety after the intervention ended.

Time-use literature commonly describes the importance of achieving both experienced and evaluative time-use satisfaction (Council, 2012). The calendar function of TLC may be associated with the caregivers’ *experience* of respite time (i.e., having (in)consistent schedule or (not) enough respite), while the coaching function may have provided the necessary support and instruction to enhance the *evaluative* dimension of time-use (i.e., ability to plan and achieve respite time-use activities that were meaningful and worthwhile). Future research should further explore how different types of activities and/or different amounts or schedules of respite time may be associated with caregiver well-being overall, as well as with caregivers’ experienced and evaluative assessments of time-use.

The staggered delivery approach afforded by our modified waitlist-control study design may have implications for intervention delivery. Caregivers exposed to TLC-Immediate effectively maintained their anxiety levels (i.e., no increase and no decrease) throughout the 16-week intervention period, while those exposed to TLC-Delayed showed a significant increase initially, followed by a marked decline once they had access to the coaching features. Those in the TLC-Delayed may have benefited from the addition of attention around week 9, when they received new instructions and access to the coaching feature. Future research can explore how caregivers used the app over time and whether engagement patterns followed exposures or reminders. Previous research and theory suggest that interventions may be more effective when there are periodic reminders or novelty built into the delivery over time to keep people engaged or to reengage them as time goes on and as use may naturally declines (Gitlin & Czaja, 2015). Building such reminders or prompts into an online intervention is relatively easy through back-end software development, and would not require significant human resources or intervention staffing to reengage participants. Elements of gamification such as badges and awards that can be earned or creating leaderboards across users are common ways to encourage continued engagement and reengagement of participants in an online intervention (Looyestyn et al., 2017).

This study focused on anxiety as a measurable dimension of caregiver well-being, given that previous research finds that anxiety is responsive to time-use interventions (Leufstadius & Eklund, 2014) and is relevant outcome for caregiving populations (del-Pino-Casado et al., 2021). Other well-being outcomes such as caregiver burden were explored using the same analytic approaches (not reported in this article); they provided similar but less significant patterns of change. Although the TLC-Delayed group showed moderate effect sizes for anxiety (an increase followed by decrease), the overall observed pattern of effect was essentially no change over the 16-week intervention exposure period. It is not uncommon to see little or no effect of interventions on caregiver well-being outcomes (Walter & Pinquart, 2020), providing an important methodological reminder to include additional measures of intervention efficacy beyond global well-being measures. In the current study, we included a variety of self-report respite time-use measures, which showed impressive and substantial changes in the quantity, quality, and overall satisfaction of their respite time and time-use. These positive effects were present for both exposure groups, suggesting that TLC, whether it was delivered in the immediate or delayed format, was likely beneficial in changing behaviors and self-perceptions about respite time-use. Yet, those effects may not be enough to offset the overall stresses associated with caregiving.

## Limitations and Future Research

The current study design did not have a traditional control group who had no exposure to the TLC intervention. It is possible that a control group would show increasing anxiety over time rather than maintenance as seen in our study, simply due to the ongoing and cumulative stresses of being a caregiver (del-Pino-Casado et al., 2021). This is a limitation of the current study design. Longitudinal data from other caregiver samples consistently show worse caregiver well-being outcomes over time, including poor physical health, heightened mental health including anxiety, and increasing reported levels of burden and strain (Adelman et al., 2014; Chiao et al., 2015; del-Pino-Casado et al., 2021; Schulz et al., 2020). The lack of statistical significance found in this sample, indicating maintenance or no change in global well-being (anxiety) over time, may well be a substantively important and meaningful outcome for this population.

A further limitation is related to the generalizability of the TLC sample, which is biased toward higher education, greater affluence, and may lack racial-ethnic diversity of the caregiver population overall. These differences may be an artifact of the types of persons who tend to be more willing to participate in intensive, controlled research studies such as this, or perhaps is reflective of demographic difference associated with “respite users” versus caregivers overall, especially since formal respite services may be cost prohibitive to some (i.e., an out-of-pocket expense for many caregivers) or may not be as prioritized to some cultural groups. The TLC intervention was codeveloped using a community advisory board, ensuring that the intervention messaging and imagery were relevant and applicable to different sociocultural groups (Utz et al., 2023), but future research should continue to explore whether and how different subgroups of caregivers engage in respite and benefit from caregiver support interventions such as TLC.

Findings from this study are likely useful in two ways—(1) as a guide for future TLC intervention refinement and dissemination, and (2) as a methodological case study, showing both a study design and analytic approach that can be applied to pilot-testing of other multicomponent interventions. Based on the findings here, future development of the TLC intervention will likely emphasize the importance of the coaching features (in addition to the calendar, dashboard, and educational resources) and periodic new features or reminders that reengage or refresh participation over time. In terms of methodological contributions, we invite other interventionists and implementation scientists to comment on and replicate the methodological approaches we used here to evaluate the initial efficacy of TLC: these included our use of a fully powered pilot sample, which allowed for an empirical evaluation of potential effect sizes, the use of a modified waitlist-control design that staggered intervention exposure in a way that isolated the key intervention features, and a statistical technique that estimated effect-size parameters associated with the timing and exposure of different intervention components, rather than the more traditional effect-size estimates associated with the passage of time. Our approach identified the features of the TLC intervention that are serving as the key mechanisms underlying the intervention’s effect, allowing for an efficient progression of TLC through the stages of intervention (re)development, testing, and implementation (Onken et al., 2014). Although the TLC study design was limited to dementia caregivers, we believe that the TLC intervention is likely applicable to other types of caregivers who use respite. Future research and implementation efforts should evaluate the effectiveness of the TLC intervention under real-world conditions and with other types of caregivers, including those who represent greater racial-ethnic, socioeconomic, and educational profiles than what was used in this pilot sample.

## Supplementary Material

igae043_suppl_Supplementary_Table_S1

## Data Availability

The study was preregistered with ClinicalTrials.gov (NCT03689179). All study materials and data for Living and Caring (TLC) study are available for public-use through the HIVE archive at the University of Utah, DOI: 10.7278/S50d-2rgg-4549. All analytic code used to generate results for this manuscript are available by request from the first author for replication purposes. A free public-use version of the TLC intervention is available at www.tlc-respite.com
