# Supplementary material for: Evaluating the Efficacy of Time for Living and Caring: An Online Intervention to Support Dementia Caregivers’ Use of Respite
Source: Innov Aging. 2024 Apr 26;8(5):igae043. doi: 10.1093/geroni/igae043 (PMC11129597; doi:10.1093/geroni/igae043)
Supplement: igae043_suppl_Supplementary_Table_S1 [file igae043_suppl_supplementary_table_s1.docx]

**Supplementary Table S1**. Demographic Characteristics of the TLC Sample (n=163), with Comparison of the TLC-Immediate (n=83)and TLC-Delayed (n=80) Subsamples

|  | **TLC Full Sample** | | **TLC-Immediate** | | **TLC-Delayed** | |  |
| --- | --- | --- | --- | --- | --- | --- | --- |
|  | n | % | n | % | n | % | *p*-value^a^* |
| **Caregiver relationship** |  |  |  |  |  |  | 0.07 |
| Spouse or partner | 113 | 69.33 | 53 | 63.86 | 60 | 75.00 |  |
| Child | 40 | 24.54 | 27 | 32.53 | 13 | 16.25 |  |
| Child's Spouse or Partner | 3 | 1.84 | 1 | 1.20 | 2 | 2.50 |  |
| Grandchild | 3 | 1.84 | 1 | 1.20 | 2 | 2.50 |  |
| Other | 1 | 0.61 | 0 | 0.00 | 1 | 1.25 |  |
| Missing/unreported | 3 | 1.84 | 1 | 1.20 | 2 | 2.50 |  |
| **Gender** |  |  |  |  |  |  | 0.85 |
| Male | 35 | 21.47 | 17 | 20.48 | 18 | 22.50 |  |
| Female | 128 | 78.53 | 66 | 79.52 | 62 | 77.50 |  |
| **Marital Status** |  |  |  |  |  |  | 0.36 |
| Married | 134 | 82.21 | 65 | 78.31 | 69 | 86.25 |  |
| Living with a partner | 5 | 3.07 | 3 | 3.61 | 2 | 2.50 |  |
| Divorced | 12 | 7.36 | 8 | 9.64 | 4 | 5.00 |  |
| Widowed | 2 | 1.23 | 2 | 2.41 | 0 | 0.00 |  |
| Never been married | 7 | 4.29 | 5 | 6.02 | 2 | 2.50 |  |
| Missing | 3 | 1.84 | 0 | 0.00 | 3 | 3.75 |  |
| **Ethnic Background** |  |  |  |  |  |  | 0.17 |
| Hispanic | 9 | 5.52 | 7 | 8.43 | 2 | 2.50 |  |
| Not Hispanic | 150 | 92.02 | 76 | 91.57 | 74 | 92.50 |  |
| Missing/unreported | 4 | 2.45 | 0 | 0.00 | 4 | 5.00 |  |
| **Race** |  |  |  |  |  |  | 0.38 |
| American Indian/Alaskan Native | 2 | 1.23 | 2 | 2.41 | 0 | 0.00 |  |
| Asian | 4 | 2.45 | 1 | 1.20 | 3 | 3.75 |  |
| Black or African American | 1 | 0.61 | 1 | 1.20 | 0 | 0.00 |  |
| Pacific Islander | 2 | 1.23 | 1 | 1.20 | 1 | 1.25 |  |
| White | 137 | 84.05 | 71 | 85.54 | 66 | 82.50 |  |
| Multiracial | 9 | 5.52 | 4 | 4.82 | 5 | 6.25 |  |
| Missing/unreported | 5 | 3.07 | 0 | 0.00 | 5 | 6.25 |  |
| **Education** |  |  |  |  |  |  | 0.93 |
| Some high school | 3 | 1.84 | 2 | 2.41 | 1 | 1.25 |  |
| High school graduate or GED | 8 | 4.91 | 4 | 4.82 | 4 | 5.00 |  |
| Some college | 60 | 36.81 | 33 | 39.76 | 27 | 33.75 |  |
| College degree | 46 | 28.22 | 22 | 26.51 | 24 | 30.00 |  |
| Graduate/Professional degree | 43 | 26.38 | 21 | 25.30 | 22 | 27.50 |  |
| Missing/unreported | 3 | 1.84 | 1 | 1.20 | 2 | 2.50 |  |
| **Income** |  |  |  |  |  |  | 0.27 |
| <$50,000 | 39 | 23.93 | 23 | 27.71 | 16 | 20.00 |  |
| >=$50,000 | 120 | 73.62 | 58 | 69.88 | 62 | 77.50 |  |
| Missing/unreported | 4 | 2.45 | 2 | 2.41 | 2 | 2.50 |  |
| **Employment** |  |  |  |  |  |  | 0.44 |
| Full-time | 41 | 25.15 | 24 | 28.92 | 17 | 21.25 |  |
| Part-time | 19 | 11.66 | 7 | 8.43 | 12 | 15.00 |  |
| Not employed | 25 | 15.34 | 14 | 16.87 | 11 | 13.75 |  |
| Retired | 75 | 46.01 | 37 | 44.58 | 38 | 47.50 |  |
| Missing | 3 | 1.84 | 1 | 1.20 | 2 | 2.50 |  |
| **Age in years** |  |  |  |  |  |  |  |
| Mean (SD) | 61.67 | (12.98) | 60.82 | (13.08) | 62.57 | (12.91) | 0.40 |
| Median (range) | 62 | (20-92) | 60 | (32-92) | 63 | (20-87) |  |
| Missing | 4 | | 1 |  | 3 |  |  |

Notes: ^a^ Mean differences were assessed with independent samples T-test, chi-square test, and Mann-Whitney U-test as appropriate. There are no statistically significant differences, *p*>.05 for all variables, suggesting baseline equivalency of the TLC-Immediate and TLC-Delayed subgroups.
